# Embedding-based alignment: combining protein language models with dynamic programming alignment to detect structural similarities in the twilight-zone

**DOI:** 10.1093/bioinformatics/btad786

**Published:** 2024-01-04

**Authors:** Lorenzo Pantolini, Gabriel Studer, Joana Pereira, Janani Durairaj, Gerardo Tauriello, Torsten Schwede

**Affiliations:** Biozentrum, University of Basel, Basel 4056, Switzerland; SIB Swiss Institute of Bioinformatics, Basel 4056, Switzerland; Biozentrum, University of Basel, Basel 4056, Switzerland; SIB Swiss Institute of Bioinformatics, Basel 4056, Switzerland; Biozentrum, University of Basel, Basel 4056, Switzerland; SIB Swiss Institute of Bioinformatics, Basel 4056, Switzerland; Biozentrum, University of Basel, Basel 4056, Switzerland; SIB Swiss Institute of Bioinformatics, Basel 4056, Switzerland; Biozentrum, University of Basel, Basel 4056, Switzerland; SIB Swiss Institute of Bioinformatics, Basel 4056, Switzerland; Biozentrum, University of Basel, Basel 4056, Switzerland; SIB Swiss Institute of Bioinformatics, Basel 4056, Switzerland

## Abstract

**Motivation:**

Language models are routinely used for text classification and generative tasks. Recently, the same architectures were applied to protein sequences, unlocking powerful new approaches in the bioinformatics field. Protein language models (pLMs) generate high-dimensional embeddings on a per-residue level and encode a “semantic meaning” of each individual amino acid in the context of the full protein sequence. These representations have been used as a starting point for downstream learning tasks and, more recently, for identifying distant homologous relationships between proteins.

**Results:**

In this work, we introduce a new method that generates embedding-based protein sequence alignments (EBA) and show how these capture structural similarities even in the twilight zone, outperforming both classical methods as well as other approaches based on pLMs. The method shows excellent accuracy despite the absence of training and parameter optimization. We demonstrate that the combination of pLMs with alignment methods is a valuable approach for the detection of relationships between proteins in the twilight-zone.

**Availability and implementation:**

The code to run EBA and reproduce the analysis described in this article is available at: https://git.scicore.unibas.ch/schwede/EBA and https://git.scicore.unibas.ch/schwede/eba_benchmark.

## 1 Introduction

Protein language models (pLMs) are becoming more popular by the day. These models capture deep “semantic relationships” between different residues in a protein by analyzing their context within the sequence, resulting in neural networks capable of generating meaningful representations at the residue level. These representations, also denoted as embeddings, are vectors in high dimensional space that can be used for a variety of downstream machine learning applications (Ferruz *et al.* 2022, Lin *et al.* 2022). Recently, pLMs were also leveraged for establishing homologous relationships between sequences. While this is achievable with standard alignment tools (Potter *et al.* 2018), whenever the comparison falls into the so-called twilight zone (Rost 1999), the pairwise signal gets blurry. This is where pLMs shine by capturing relationships far beyond simple sequence comparisons, uncovering otherwise undetected evolutionary relationships that can guide, for example, protein annotation or structure prediction efforts.

**Figure 1.**
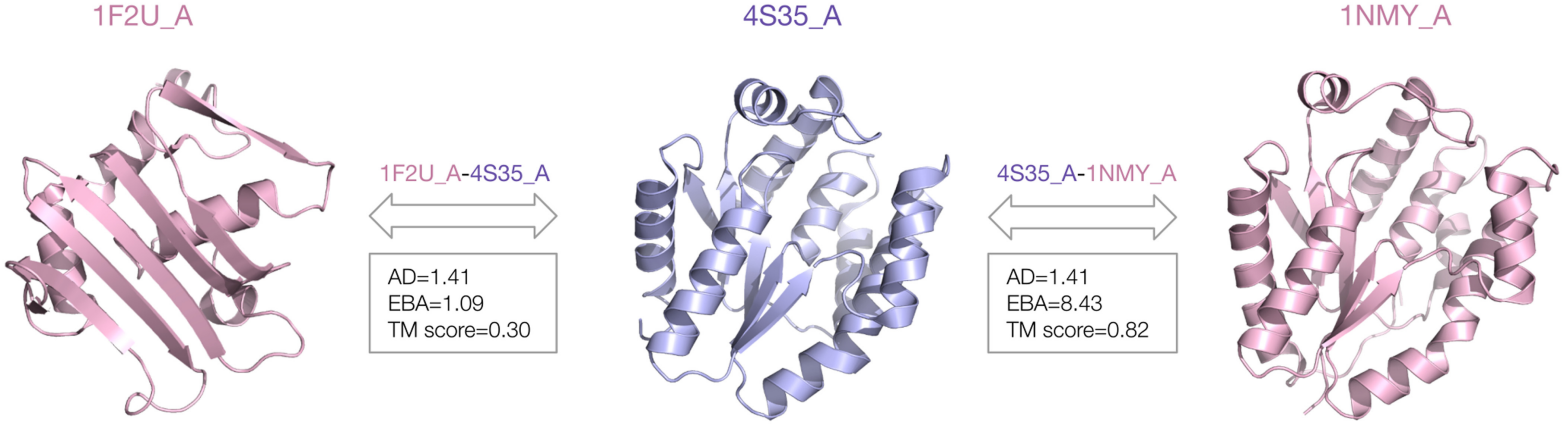
Comparison of three proteins—Thymidylate kinase from *Aquifex Aeolicus* VF5, human thymidylate kinase, and Rad50 ATPase from *Pyrococcus furiosus* (PDB ids: 4S35, 1NMY, and 1FU2 chain A)—using sequence and structure based scores. While 4S35 and 1NMY share a very similar fold (TM-score = 0.82), the 1F2U structure is different (TM-score = 0.30). However, the average representation of the protein sequence in the center (PDB id: 4S35) has approximately the same distance in the embedding space (AD = 1.41) to both other proteins. In this example, the AD is not able to distinguish the two cases, while our EBA assigns a much higher score to the pair of sequences with a similar fold. The proteins in this example share low sequence identity: 15% for 1F2U-4S35 and 29% for 4S35-1NMY. Both the AD and EBA scores in this example were computed using the ProtT5 language model and the Euclidean distance metric.

For detecting such relationships with pLMs, protein sequences are commonly projected into an high-dimensional space by averaging their per-residue embeddings (Heinzinger *et al.* 2022, Hie *et al.* 2022, Schütze *et al.* 2022). However, the meaning of distance in this space is still unclear. In Hie *et al.* (2022), the Euclidean distance in the averaged embedding space was used to quantify sequence similarity, which in turn was used to generate an evolutionary landscape of homologous proteins by connecting sequences to their k-nearest neighbors. The distance between the average representations was used again in Heinzinger *et al.* (2022) to establish distant homology relationships between CATH domains (Sillitoe *et al.* 2020). Performance was improved by contrastive learning to re-project the average embedding representation into a space where similar CATH domains cluster closely together. A similar approach was adopted by Hamamsy *et al.* (2023) to build TM-vec, a tool able to predict TM-scores. However, representing a sequence by averaging its per-residue embeddings has limitations. An example is given by multidomain proteins, where a loss of signal can be expected when averaging per-residue embeddings from distinct domains that potentially evolved independently (Schütze *et al.* 2022). Furthermore, even for single domain sequences, average-based similarity metrics inherently lose information on order and are affected by comparisons of residues without any evolutionary relationship. An example is shown in Fig. 1, where the average representation of a sequence is equidistant from a protein with the same structure and another one with a completely different fold.

These problems can be alleviated by methods constructing explicit alignments at the cost of added computational complexity. Two examples of embedding-based alignment (EBA) methods were introduced by Bepler and Berger (2021) and Hamamsy *et al.* (2023). In Bepler and Berger (2021), a language model was trained using both sequence and structural information. They showcase a “soft alignment” generated with a weighted sum of all the possible pairwise residue distances. The resulting score predicted homologous relationships between SCOP (Andreeva *et al.* 2019) domains. On the other hand, in Morton *et al.* (2020), a network fed with residue embeddings was trained on protein structures to generate dynamic alignment parameters, such as the score and gap penalty matrices.

In our work, we introduce an EBA that, given two sequences, leverages the distance of all possible pairs of residue embeddings to generate a “similarity matrix” that is then used as a score matrix in a classical dynamic programming alignment. As we observed that residue-level embeddings are not always comparable across sequences, we also include a step to enhance the signal of the similarity matrix using the distributions of residue embedding distances of the compared proteins (Supplementary Fig. S6). The score obtained with the alignment is able to capture structural similarity even in the sequence-similarity twilight zone (Rost 1999), outperforming other pLM methods and classic sequence-based approaches in the detection of distant homologies. Such an approach allows the generation of reliable protein sequence and structure alignments at low sequence similarity, opening the door to the annotation and interpretation of protein sequences without clear homologs of known structure and function.

The idea behind our approach is similar to Hamamsy *et al.* (2023), however, the absence of any training and parameter optimization makes our method robust to generalization and easy to interpret. Furthermore, the method is not bound to a specific language model, therefore any pLM can be utilized, leaving a choice based on the requirements of specific scientific applications. Another similar method is pLM-BLAST (Kaminski *et al.* 2023), which was developed at the same time as our method. However, we demonstrate that our proposed signal enhancement method can greatly improve the performance of such language model embedding alignment approaches.

## 2 Materials and methods

The methods described in this section rely on the assumption that residues with similar characteristics and context will have similar embeddings, therefore, they will be close in the embedding space. Benchmarks have been performed with three pretrained state of the art pLMs: ProstT5 (Heinzinger *et al.* 2023), ProtT5-XL-UniRef50 (ProtT5) (Elnaggar *et al.* 2022), and esm1b_t33_650M_UR50S (ESM-1b) (Rives *et al.* 2021). Both ProtT5 and ESM-1b are based on the transformer architecture (Vaswani *et al.* 2017) and trained on UniRef50 (Suzek *et al.* 2014) in a self-supervised fashion to predict masked amino acids. ProstT5 is based on ProtT5 and encodes both sequence and structural information by leveraging the 3Di-tokens generated by Foldseek (van Kempen *et al.* 2023). The residue-representations generated with these models are vectors belonging to spaces with 1024 (ProstT5, ProtT5) and 1280 (ESM-1b) dimensions. It has been shown that, based on their position in the embedding space, amino-acids can be clustered according to biochemical and biophysical properties (Rives *et al.* 2021, Elnaggar *et al.* 2022).

### 2.1 Average distance

Averaging per-residue embeddings is a simple and widely used approach to derive a fixed size representation for sequences of variable length (Heinzinger *et al.* 2022, Hie *et al.* 2022, Schütze *et al.* 2022). Once the sequences are projected in this fixed size space, it is possible to compute the distance between them; we refer to this approach as the average distance (AD) method. Any distance metric can be used for this purpose and in this work, driven by preliminary analysis, we use Euclidean distances. AD is computationally efficient and captures meaningful relationships between proteins (Bepler and Berger 2021, Hie *et al.* 2022, Schütze *et al.* 2022).

### 2.2 Embedding-based alignment

EBA aims to fully utilize the information encoded in the per-residue embeddings provided by pre-trained language models. Two protein sequences are compared by constructing a similarity matrix, which is used as a score matrix to build an explicit alignment. The alignment score is finally used to define protein similarity. Given two sequences *A* and *B*, with lengths *n* and *m*, respectively, the per-residue embedding similarity matrix SM_*n*__×__*m*_ is built by computing the similarity score SM_*i*__,__*j*_ for each possible pair of residues:

(1) $${\mathrm{SM}}_{i,j}=e^{-d(r_{i},r_{j})},$$

with d(): the desired distance metric, $r_{i}$: embedding of residue i∈A, $r_{j}$: embedding of the residue j∈B. All the analysis in this work were performed using Euclidean distance as d().

#### 2.2.1 Signal enhancement

The signal in the similarity matrix is enhanced by comparing the similarity of each pair of residues with the scores of all pairs involving the amino-acids of the two proteins under consideration. Given a pair of residues (*i*, *j*) with a similarity score ${\mathrm{SM}}_{i,j}$, we compute the Z-score with respect to both the elements in the same row (${\mathrm{SM}}_{i,*}$) and column (${\mathrm{SM}}_{*,j}$). We finally convert each element of the similarity matrix to the average of the computed Z-scores.

(2) $$x_{r}(i)=\frac{1}{m}\sum_{k}^{m}e^{-d(r_{i},r_{k})}\;\sigma_{r}(i)=\sqrt{\frac{1}{m}\sum_{k}^{m}{\left(e^{-d(r_{i},r_{k})}-x_{r}(i)\right)}^{2}},$$

(3) $$x_{c}(j)=\frac{1}{n}\sum_{k}^{n}e^{-d(r_{k},r_{j})}\;\sigma_{c}(j)=\sqrt{\frac{1}{n}\sum_{k}^{n}{\left(e^{-d(r_{k},r_{j})}-x_{c}(j)\right)}^{2}},$$

with $x_{r/c}(i/j)$ and $\sigma_{r/c}(i/j)$ being the average and standard deviation computed for the row/column *i*/*j*.

(4) $$z_{r}(i,j)=\frac{e^{-d(r_{i},r_{j})}-x_{r}(i)}{\sigma_{r}(i)}\;z_{c}(i,j)=\frac{e^{-d(r_{i},r_{j})}-x_{c}(j)}{\sigma_{c}(j)}.$$

Each element of the enhanced similarity matrix is then computed as:

(5) $${\mathrm{SM}}_{\mathrm{enh}(i,j)}=\frac{z_{c}(i,j)+z_{r}(i,j)}{2}.$$

#### 2.2.2 Global and local dynamic alignment

Both Needleman–Wunsch (NW) and a Smith–Waterman (SW) were implemented using ${\mathrm{SM}}_{\mathrm{enh}}$ as score matrix. Gap penalties were set to 0 for NW and to 2 for SW. Furthermore, we subtracted a constant, K=2, to ${\mathrm{SM}}_{\mathrm{enh}}$ for the SW implementation used in the example showcased in Section 4.6. For NW global alignment, the EBA similarity score is normalized as follows: given the resulting alignment score *s*_align_, the EBA similarity score is defined as:

(6) $${\mathrm{EBA}}_{\min/\max}=\frac{s_{\mathrm{align}}}{l_{\max/\min}},$$

where $l_{\min/\max}$ is the length of the shorter/longer sequence involved in the comparison. The alignment score $s_{\mathrm{align}}$ is symmetric with respect to the sequences $s_{\mathrm{align}}(A,B)=s_{\mathrm{align}}(B,A)$. The symmetry is broken after normalization according to the length of one of the two sequences, similarly to the normalization adopted for the computation of TM-score for structure comparison (Zhang and Skolnick 2004).

## 3 Benchmark

### 3.1 Structural similarity analysis

We benchmarked AD and EBA in capturing structural similarities in the absence of clear sequence similarity. For that, we gathered protein pairs of known structure with low sequence identity using PISCES (Wang and Dunbrack 2003) (default parameters, with the exception of: “Maximum pairwise percent sequence identity”: 30% and “Minimum chain length”: 75). The resulting 19 599 pairs exhibit detectable homology (Hhsearch, Steinegger *et al.* 2019, *e*-value threshold $10^{-4}$) but are only remotely related (sequence identity <30%). Performances of EBA and AD were measured as Spearman correlation coefficients between the predicted similarity/distance and structural similarity, expressed as the TM-score (Zhang and Skolnick 2005).

### 3.2 CATH annotation transfer analysis

To assess EBA capabilities for transferring CATH domain annotations, we used the lookup and test set from Heinzinger *et al.* (2022). In Heinzinger *et al.* (2022), annotations from a lookup set of 66K CATH domains were transferred to a test set of 219 elements. As described in ProtTucker (Heinzinger *et al.* 2022), the lookup set was built making sure that the sequence similarity to the test set is very low (HVAL < 0, Rost 1999) and that for each element in the test set at least one sequence with an identical label can be found in the lookup set. Our number of matching domain annotations between the test set and the lookup set agrees with the ProtTucker number except for three missing cases in the Topology category. Given a domain in the test set, the annotation of the domain with the higher EBA score across those in the lookup set is transferred. We carried out this analysis for each of the four CATH categories using EBA. We computed the accuracy of the annotation transfer as:

(7) $$\mathrm{Accuracy}(y,\hat{y})=\frac{1}{n_{\mathrm{samples}}}\sum_{i}^{n_{\mathrm{samples}}}1(y=\hat{y}).$$

Results were compared to the scores reported in Heinzinger *et al.* (2022) for AD, ProtTucker (Heinzinger *et al.* 2022), and HMMER (Potter *et al.* 2018) and the ones reported in Hamamsy *et al.* (2023) for TMvec, Foldseek, and MMseqs2.

### 3.3 SCOP annotation transfer analysis

To assess EBA performances in transferring SCOP annotations, we used the dataset illustrated in van Kempen *et al.* (2023). Protein domains in SCOPe 2.01 (Andreeva *et al.* 2019) were clustered at 40% sequence identity, resulting in 12, 211 non-redundant domains: SCOPe40. The clustered sequences were retrieved from https://github.com/steineggerlab/foldseek-analysis. We used EBA for scoring all possible pairs of domains within this data set, with the goal of identifying sequences belonging to the same family, super family and fold. Then, for each query, we computed the sensitivity up to the first false-positive, defined as a match to a different fold. Our results were compared to the scores reported in van Kempen *et al.* (2023) for the following methods: Foldseek, Foldseek-TM, DALI (Holm and Sander 1993), CLE-SW (Wang and Zheng 2008), and MMseqs2 (Steinegger and Söding 2017). With the exception of MMseqs2, these methods rely on structural information.

### 3.4 HOMSTRAD alignment quality

To assess alignment quality we used the HOMSTRAD database (Mizuguchi *et al.* 1998). HOMSTRAD encompasses expertly curated structural alignments of homologous proteins within 1032 protein families. We reproduced the analysis performed in van Kempen *et al.* (2023) and compared our results to: Foldseek, Foldseek-TM, DALI, CLE-SW and MMseqs2. Again, the data were retrieved from https://github.com/steineggerlab/foldseek-analysis. For each family, the pairwise alignment of the first and last member was collected, resulting in 1032 pairs of sequences. Using the HOMSTRAD alignments as ground truth, we computed both sensitivity and precision for the aligned residues in each pair and averaged the results across the families.

(8) $$\mathrm{sensitivity}=\frac{\mathrm{tp}}{\mathrm{tp}+\mathrm{fn}}\;\;\mathrm{precision}=\frac{\mathrm{tp}}{\mathrm{tp}+\mathrm{fp}},$$

with tp/fp being the number of true/false positives and fn the number of false negatives.

## 4 Results

### 4.1 EBA captures structural similarity in the twilight zone

We benchmarked EBA, as described in Section 3.1, against the following methods:

- EBA without signal enhancement (EBA_plain_) (Section 2.2.1)
- Average distance (Section 2.1)
- ProtTucker (Heinzinger *et al.* 2022)
- TM-vec (Hamamsy *et al.* 2023)
- pLM-BLAST (Kaminski *et al.* 2023)
- HHalign (Steinegger *et al.* 2019)
- Needleman–Wunsch with a BLOSUM matrix, normalized sequence similarity

We compared the predicted similarities/distances to the TM-scores computed with TM-align (Zhang and Skolnick 2005). The asymmetric nature of TM-score allows to perform the analysis using both the score normalized by the length of the longer or the shorter sequence, ${\mathrm{TM}}_{\min}$ and ${\mathrm{TM}}_{\max}$, respectively. The choice of the score depends on the type of similarity one would like to investigate. The scores reported in Table 1 show the Spearman correlation computed using both ${\mathrm{TM}}_{\min}$ and ${\mathrm{TM}}_{\max}$, normalizing the EBA score consistently with the TM-score normalization.

**Table 1.** Spearman correlations between the similarity/distance predictions of the listed methods and TM scores.^a^

	TM_min_			TM_max_		
	ProstT5	ProtT5	ESM-1b	ProstT5	ProtT5	ESM-1b
EBA	**0.92**	0.90	0.87	**0.86**	0.84	0.80
EBA_plain_	0.56	0.72	0.64	0.20	0.54	0.52
TM-vec		0.81			0.82	
pLM-BLAST		0.58			0.60	
AD	−0.65	−0.46	−0.46	−0.49	−0.39	−0.39
ProtTucker		−0.46			−0.38	
HH-align	0.82			0.77		
Needleman–Wunsh	0.61			0.43		

a Where possible, we showcase the methods performances for ProstT5, ProtT5 and ESM-1b. The EBA scores are normalized according to the TM scores, we therefore compare EBA_min_ with TM_min_ and EBA_max_ with TM_max_. Since the other methods provide only one score, the same prediction is compared to both TM_min_ and TM_max_. The expected correlation for similarity scores is positive, while for distances is negative. Best correlation values for TM_min/max _in bold.

Our results indicate that EBA outperforms all other approaches, independently of the underlying language model. Table 1 shows that the signal enhancement step significantly contributes to the performance of EBA by comparing it to the version based on raw similarity scores: EBA_plain_. The strong impact of the signal enhancement suggests that raw distances, and thus the similarity scores, need to be contextualized as they are not necessarily comparable among different pairs of sequences. This is done by substituting the raw similarity of each residue pair with its pseudo Z-score, as described in Section 2.2.1. This approach extracts the signal by assigning high values to residue pairs with high similarity with respect to other pairs involving the same residues. A comparison worth mentioning is the one with pLM-BLAST, which aligns based on cosine similarity of residue pLM embeddings. While here we show the results of pLM-BLAST-local which may explain it’s particularly poor performance when correlated to a global measure such as the TM-score, the results from their benchmarking effort (Kaminski *et al.* 2023) still revealed that EBA outperforms pLM-BLAST-global for homology detection in the twilight zone. Also interesting is the comparison with a classical NW alignment performed using a BLOSUM matrix (Henikoff and Henikoff 1992). Since the alignment algorithm is the same as in EBA, here we are directly comparing our similarity matrix to a classical BLOSUM matrix. The fact that this method is outperformed even by EBA_plain_ highlights the highly informative nature of the embedding distances. Details concerning the methods used as comparison can be found in (Supplementary Section S1).

### 4.2 Length normalization

**Figure 2.**
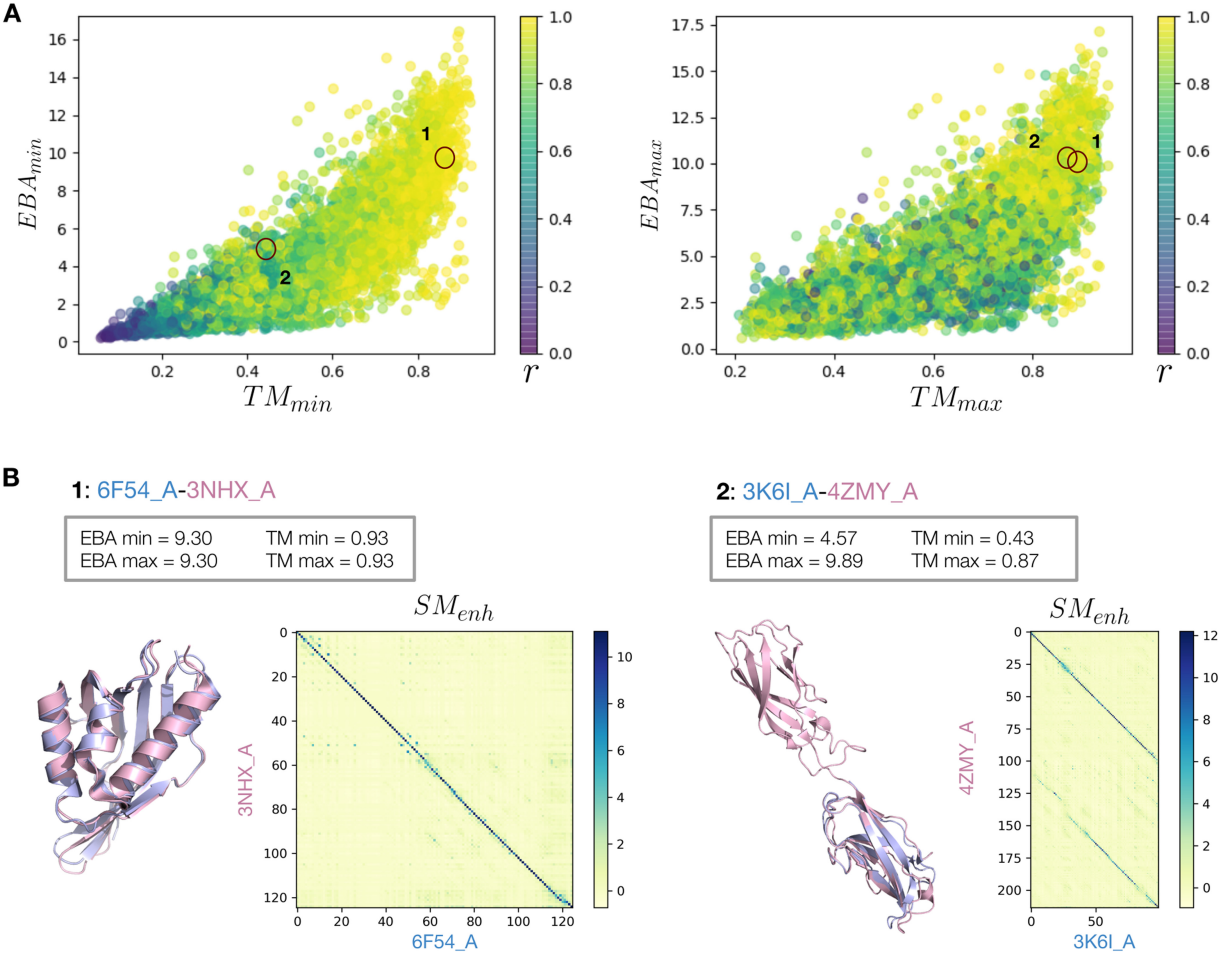
(A) The correlation between EBA_min/max_ and TM_min/max_ for the analysis performed using ProtT5. A color gradient shows the length ratio of the sequence pairs: *r* = *l*_min_/*l*_max_. Where *l*_min_ is the length of the shorter sequence and *l*_max_ the length of the longer one. (B) Two pairs of sequences with very different *r*. Pair one is not affected by the length normalization, while pair 2 score changes drastically between EBA_min_ and EBA_max_.

The estimation of similarity between two proteins is affected by their difference in length. Whenever this difference is large, the choice of the normalization becomes an important factor. An example is shown in Fig. 2, where we consider a pair of sequences with the same length (pair 1) and a second pair in which one sequence is approximately double the size of the other (pair 2). In the first example, since the sequences have the same length, the normalization is irrelevant: ${\mathrm{TM}}_{\min}=TM_{\max}$ and EBA${}_{\min}$ = EBA${}_{\max}$. In the second pair on the other hand, the shorter protein is entirely contained in the longer one. In this case, EBA${}_{\min}$ and EBA${}_{\max}$ offer two different perspectives. The normalization according to the shorter sequence results in a large score (EBA${}_{\max}=9.54$), reflecting the fact that the shorter sequence successfully aligned through its whole length. However, the longer sequence is only partially aligned, therefore the normalization according to its length results in a lower score (EBA${}_{\min}=4.41$). We observed that in all the annotation transfer analysis the best performances are obtained by normalizing the EBA score by the length of the longer sequence in the comparison. We therefore suggest using EBA${}_{\min}$ for this kind of analysis.

### 4.3 EBA successfully transfers CATH annotations

Table 2 shows CATH annotation transfer performance of EBA vs. other sequence-based, pLM-based and structure-based methods. ProtTucker, which is trained and optimized for this task, outperforms AD for the ProtT5 pLM. However, EBA on ProtT5 surpasses this performance for both topology (T) and homology (H) label transfer, despite not relying on training or parameter optimization for any specific task. EBA with ProstT5 as the underlying pLM offers the best performances overall across all these methods, surpassing both classic sequence profile based tools and Foldseek, which relies on structural information. As normalization for this analysis, we used the length of the longer sequence in each comparison, therefore EBA${}_{\min}$. With this normalization, selecting the higher similarity scores ensures to value both similarity and sequence coverage in the comparison.

**Table 2.** Accuracy computed for the CATH annotation transfer analysis as in Heinzinger *et al.* (2022).^a^

	ProstT5			ProtT5					ESM-1b			Not pLMs-based		
	EBA	EBA_plain_	AD	EBA	EBA_plain_	ProtTucker	TM-vec	AD	EBA	EBA_plain_	AD	Foldseek	HMMER	MMseqs2
C	**91**	79	86	87	82	88	89	84	89	75	79	77	70	53
A	**84**	66	75	77	68	77	80	67	78	59	61	73	60	33
T	**78**	55	63	74	63	68	71	57	70	54	50	59	59	21
H	**88**	61	67	85	74	79	81	64	77	61	57	77	77	25

a The reported EBA and EBA_plain_ values are normalized according to the length of the longer sequence in each comparison: EBA_min_. Best performance for each CATH category in bold.

### 4.4 EBA competes with structure-based methods

**Figure 3.**
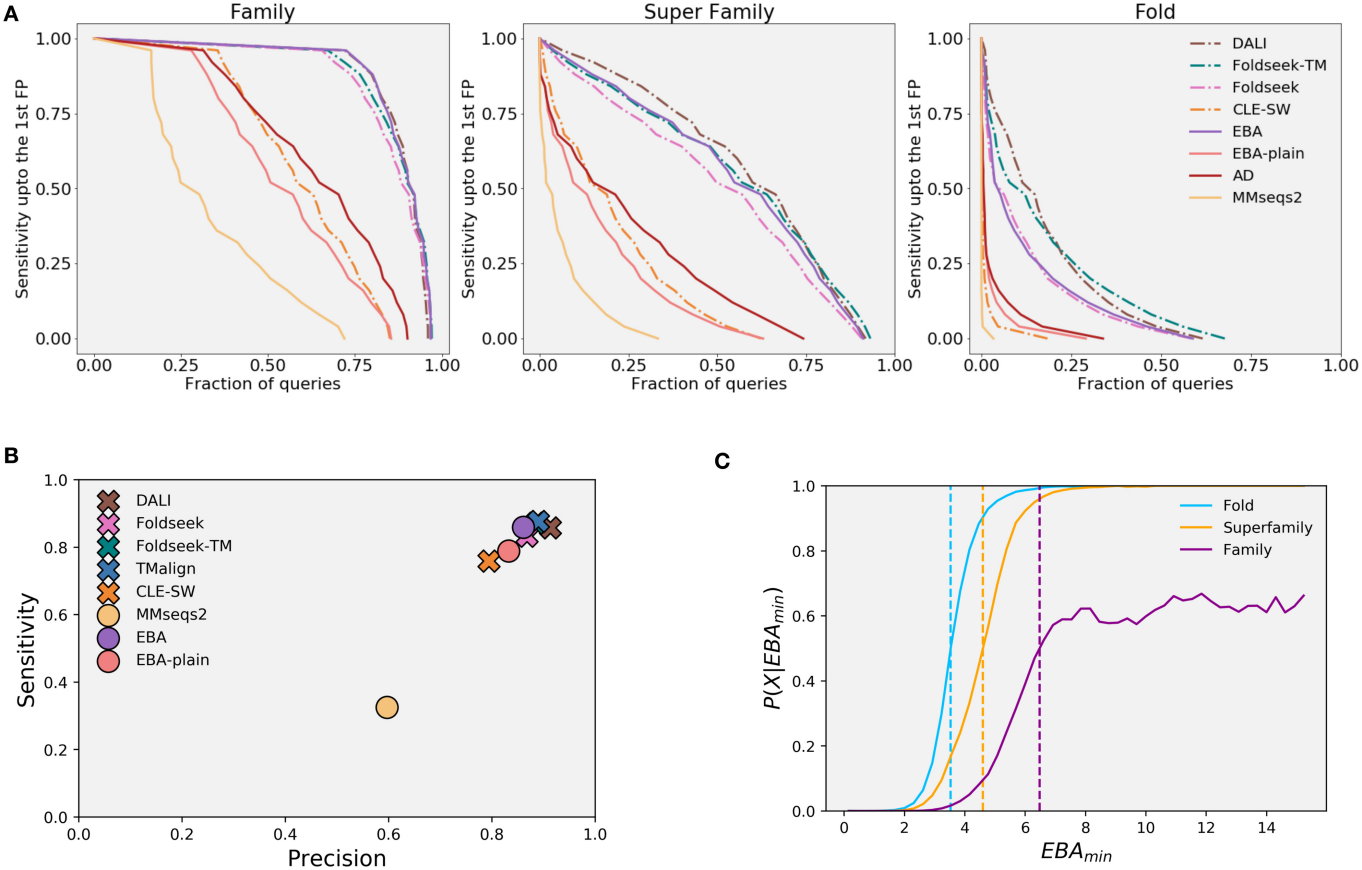
Cumulative sensitivity distribution for the annotation transfer analysis on the SCOPe40 dataset for: family, superfamily, and fold. The sensitivity is computed as the area under the ROC curve up to the first FP. With TPs being matches within the same group and FPs being matches between different folds. The reported score is EBA_min_ with ProstT5 as underling language model. Structure-based methods are shown with a dash-dot line and sequence/pLM-based ones are shown with a continuous line. (B) Alignment quality expressed as sensitivity versus precision for the HOMSTRAD benchmark. With sensitivity being: TP residues in alignment/query length and precision being TP residues/alignment length. Structure-based methods are marked with an x, while the sequence/pLM-based ones are marked with a dot. (C) Posterior probability of belonging to the same group in the annotation transfer analysis on the SCOPe40 database. The posterior probabilities are again computed using EBA_min_ with ProstT5 as underling language model.

As described in Sections 3.3 and 3.4, we benchmarked EBA against state of the art structure-based methods for annotation transfer and alignment quality. Consistently with the other benchmarks in this paper, the best performer in the SCOP annotation transfer analysis is EBA${}_{\min}$-ProstT5. While the three pLMs have relatively similar performances in this analysis (Supplementary Fig. S3), EBA${}_{\min}$-ProstT5 increasingly has an edge over the other two in the classification of superfamilies and folds. This is not too surprising since ProstT5 includes structural information despite needing only sequence as input. Notably, EBA and DALI are the best performers in terms of family annotation transfer, closely followed by Foldseek and Foldseek-TM (Fig. 3A). The performances of EBA slightly drops in the superfamily classification, where it performs as good as Foldseek-TM, and drops again in the fold classification, in which it offers results similar to Foldseek. Notice that Foldseek-TM uses TM-align to re-align high-scoring hits generated with Foldseek.

In the alignment quality benchmark, consistently with the other analysis, the signal enhancement improves the performances with respect to EBA_plain_. EBA-ProstT5, outperforms EBA-ProtT5 and EBA-ESM-1b and as Fig. 3B shows, EBA-ProstT5 has similar performances to the best structure-based methods: DALI, TM-align, Foldseek-TM, and Foldseek, with a slight disadvantage compared to the best structural aligners in precision, and an advantage compared to Foldseek in recall (Supplementary Table S4). A direct comparison of the F1 scores of the alignments generated with EBA, DALI, and Foldseek for each HOMSTRAD family can be found in Supplementary Fig. S5. Overall, EBA performances are at the level of structure-based methods. Nowadays having structural information is easier thanks to AlphaFold (Jumper *et al.* 2021), however having good models is not always a given. Furthermore, EBA offers an evolutionary perspective together with the structural one, and allows the comparison of proteins with disordered regions. The main drawback compared to very fast methods like Foldseek and MMseqs2 is the computation time, which with the current implementation averages a comparison every 0.02 s on a CPU assuming pre-computed embeddings (Supplementary Section S2). Uncompressed storage of these embeddings requires 4⋅l⋅N bytes with *l* being sequence length and *N* the pLM specific embedding dimension. In the example of ProstT5, this requires 7.5GB to store all of SCOPe40. However, storage requirements can be avoided by computing embeddings on the fly, which on average, takes around 0.02 s per protein sequence on GPU, which is much faster than structure prediction.

### 4.5 The meaning of EBA scores

We quantify the meaning of EBA scores by estimating Bayesian posterior probabilities in SCOPe40. We performed the analysis on EBA${}_{\min}$ scores computed with ProstT5 for all sequence pairs in SCOPe40. The posteriors are used as a certainty measure of being in the same fold, superfamily or family given an EBA${}_{\min}$ score and are defined as:

(9) $$P(X|{\mathrm{EBA}}_{\min})=\frac{P({\mathrm{EBA}}_{\min}|X)P(X)}{P({\mathrm{EBA}}_{\min}|X)P(X)+P({\mathrm{EBA}}_{\min}|\bar{X})P(\bar{X})}.$$

With *X* representing the event of two domains belonging to the same fold, superfamily, or family, $\bar{X}$ the complementary event, P(X)/$P(\bar{X})$ the prior probability associated to that event and $P({\mathrm{EBA}}_{\min}|X)$/$P({\mathrm{EBA}}_{\min}|\bar{X})$ the likelihood of EBA${}_{\min}$ given *X*/$\bar{X}$. As EBA${}_{\min}$ has no upper bound, data points corresponding to the top 0.01% scores have been removed from the analysis. This gives prior probabilities of 0.0082, 0.0036, and 0.0009 for being in the same fold, super family and family respectively. A steady shift toward higher EBA${}_{\min}$ scores can be observed for the posterior distributions when going from fold toward family in the SCOP hierarchy (Fig. 3C). EBA${}_{\min}$ scores with posterior probability >0.5 indicate that two sequences are related at a given hierarchy level with a reasonable certainty which leads to an EBA${}_{\min}$ threshold of 3.5 for fold, 4.6 for super family, and 6.5 for family. These values are specific for the ProstT5 pLM, and posterior distributions for other pLMs are available in (Supplementary Fig. S2).

### 4.6 Domain permutation detection

**Figure 4.**
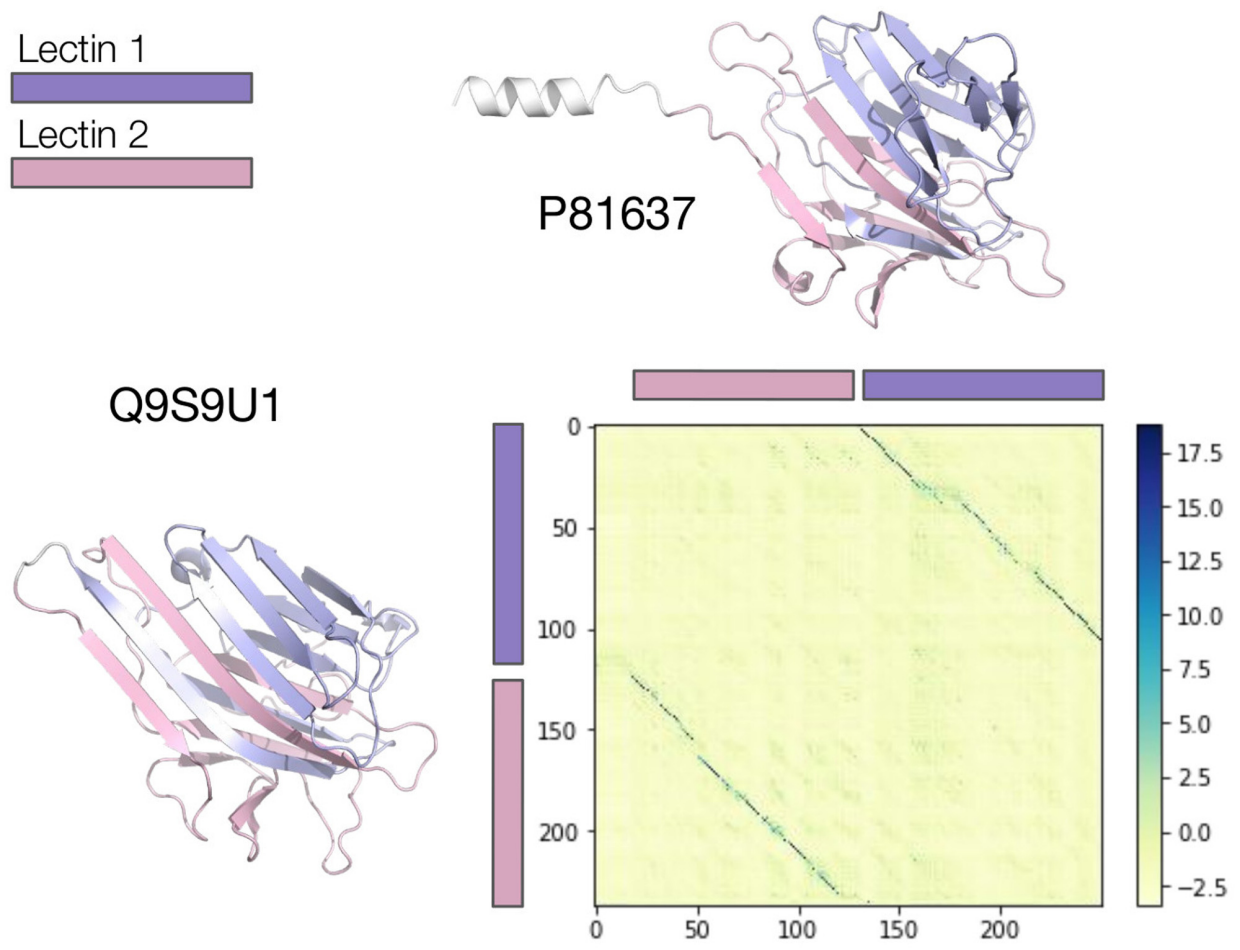
Similarity matrix for the comparison of Q9S9U1 and P8163, indicated in BaliBase 2 as: LECA DIOGU and AAD48977, respectively. These two sequences share 2 Lectin domains, arranged in a different order. For this figure, we truncated P81637 to include exclusively the part of the similarity matrix containing the shared domains. The displayed structures were downloaded from the AlphaFold Protein Structure Database (Varadi *et al.* 2021).

The similarity matrix described in Section 2.2 can also be used to generate local alignments, unlocking applications such as domain annotation or the identification of circular permutations. As a proof of concept, we implemented a SW local alignment with our similarity matrix as input. We used it on an example from the BaliBase2 database (Bahr *et al.* 2001) exhibiting a circular permutation where the two domains have modest sequence similarity (19% and 26% sequence identity). Notably, our local alignment correctly maps the domains as depicted in Fig. 4. The presence of gap penalties and negative scoring values for residues mismatches is important for the correct behavior of the local alignment. In principle, parameters optimization could lead to further improvements of our approach for specific applications, but we leave this open for future exploration.

## 5 Conclusions

In this work, we showcase the potential of combining pLM representations and classical alignment methods for establishing distant homology relationships. Our EBA approach is able to identify structural similarities between proteins in the twilight zone, where pairwise sequence identity goes far below 30%. Our results indicate that, in such applications, pLM-based score matrices are a more robust option when compared to classic alternatives. This may be due to the ability of pLMs to capture not only residue biochemical characteristics but also their context in the full proteins. Despite the absence of additional training or parameter optimization, EBA outperforms other state of the art pLM-based methods, classical approaches and even structure-based tools (Table 1). The absence of any sort of re-training and optimization not only makes the approach extremely generalizable but also allows to leverage different pLMs, which makes it adaptable to this fast evolving field. This was demonstrated by the inclusion of the recently released ProstT5 in our analysis without the need of any algorithmic modification. Interestingly, among the benchmarked pLMs, ProstT5 is the one that benefits the most from our proposed Z-score-based signal enhancement, which boosts EBA-ProstT5 performance to even surpass structure-based methods. EBA computation times are higher than the fast average-based methods and highly optimized tools like MMseqs2 and Foldseek. While still reasonably fast for the alignment or comparison of well-defined sets of sequences, this may become limiting for very large-scale analyses. One way to overcome this is to carry out a pre-filtering step by first identifying putative close sequences using AD and then a higher-resolution alignment with EBA, which has a better time complexity than TM-align (Supplementary Fig. S1). This optimization approach was also proposed in similar works, such as: Hamamsy *et al.* (2023) and Kaminski *et al.* (2023).

In our work, we generated a pairwise alignment, however, the same score matrix (${\mathrm{SM}}_{\mathrm{enh}}$) can be employed for a multiple sequence alignment (MSA). This would allow for the construction of MSAs involving highly divergent and dissimilar sequences, providing, for example, better inputs for deep learning methods that rely on MSAs, such as AlphaFold (Jumper *et al.* 2021). Recently, a pLM-based MSA method was proposed by McWhite and Singh (2022). Here, the authors generate MSAs by clustering and ordering amino acid contextual embeddings.

The rising popularity of EBA methods (McWhite and Singh 2022, Hamamsy *et al.* 2023, Kaminski *et al.* 2023) highlights their potential. We believe that the rapid development of such methods will soon further transform protein bioinformatics; opening new doors into the modeling and annotation of proteins, beyond the detection horizon of current state-of-art tools.

## Supplementary Material

btad786_Supplementary_Data

## Data Availability

The code to reproduce the analysis described in this article is available at: https://git.scicore.unibas.ch/schwede/EBA and https://git.scicore.unibas.ch/schwede/eba_benchmark. The repository also contains detailed instruction on how to generate the enhanced similarity matrix for a pair of sequences and score them with the EBA method.
