# Supplementary material for: Embedding-based alignment: combining protein language models with dynamic programming alignment to detect structural similarities in the twilight-zone
Source: Bioinformatics. 2024 Jan 4;40(1):btad786. doi: 10.1093/bioinformatics/btad786 (PMC10792726; doi:10.1093/bioinformatics/btad786)
Supplement: btad786_Supplementary_Data [file btad786_supplementary_data.pdf]

Supplementary material for: Embedding-based alignment: combining protein language models with dynamic programming alignment to detect structural similarities in the twilight-zone

This supplementary material accompanies the paper titled "Embedding-based alignment: combining protein language models with dynamic programming alignment to detect structural similarities in the twilight-zone". This document provides additional details regarding the methods compared to EBA in section 3.1. Here we also show the computation times for the same analysis. Furthermore, we describe an example that shows how our method handles domain flexibility.

1. METHODS DETAILS

Needleman-Wunch with BLOSUM matrix

We run the Needleman-Wunch global alignment using EMBOSS Needle [7] with default parameters: "score matrix": EBLOSUM62, "Gap_penalty": 10.0, "Extend_penalty": 0.5. Here we report the following Needle output: alignment score, sequence identity and sequence similarity. Alignment score has been normalized the same way as the EBA alignment score. Best performances are obtained using sequence similarity and are reported in Table 1 of the main manuscript.

Table S1. Spearman correlations between the similarity predictions obtained running a Needleman-Wunch global alignment. The best scores on this table are reported in Table 1 of the manuscript.

	TM_{min}	TM_{max}
Sequence Similarity	**0.61**	**0.43**
Sequence Identity	0.60	0.43
Alignment Score	0.56	0.41

HHalign

We generated alignments for the Pisces's pairs using HHalign [10] with default parameters. The required profiles were generated with two iterations of HHblits [10] with default parameters on UniClust30 30_2018_08. We normalized the alignment score produced by HHalign as in EBA, by dividing by the length of the longer/shorter sequence for the comparison to TM_{max}/TM_{min}, respectively.

TM-vec

We installed TM-vec [2] version 1.01 from https:https://github.com/tymor22/tm-vec. We then performed the analysis with each one of the 4 TM-vec models available: 'tm_vec_swiss_model.ckpt', 'tm_vec_swiss_model_large.ckpt', 'tm_vec_cath_model_.ckpt' and 'tm_vec_cath_model_large.ckpt'. The best performances are obtained using 'tm_vec_cath_model_large.ckpt', and are reported in Table 1 of the manuscript. Since TM-vec predicts TM scores no normalization is needed.

Table S2. Spearman correlations between the similarity predictions obtained running TM-vec. The best scores (cath model large) are reported in Table 1 of the manuscript.

	TM_{min}	TM_{max}
swiss model	0.78	0.70
swiss model large	0.77	0.75
cath model	0.80	0.79
cath model large	**0.81**	**0.82**

pLM-BLAST

We installed pLM-BLAST from https://github.com/labstructbioinf/pLM-BLAST on date 27/12/2022. Since pLM-BLAST [5] generates a local alignment, multiple alignments are associated to each pair of sequences. For each pair of sequences in the PISCES data, we selected the best alignment score as a proxy for sequence similarity.

ProtTucker

We downloaded the pre-computed embeddings for the PDB sequences from: https://github.com/Rostlab/EAT. We then selected the embeddings associated to the sequences used in the analysis 3.1 of the manuscript. Finally, we computed the Euclidean distance between these embeddings.

2. EBA COMPUTATION TIME

We report here the EBA computation times for the sequence pairs of the analysis described in section 3.1 of the manuscript. Each point represents a pair of sequences. We can observe how the time needed to compute the EBA score grows linearly with the product of the length of the sequence pair. These analysis were run on a single thread on a "AMD EPYC 7742 64-core" Processor.

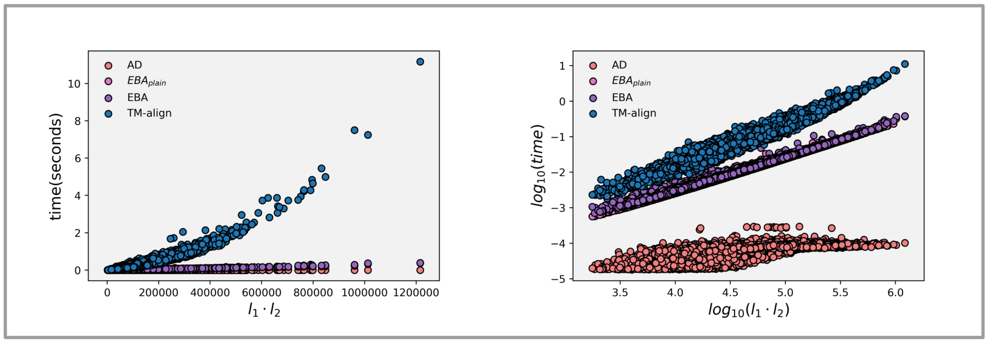

Fig. S1. Computation times for PISCES's pairs in function of the product of the sequences length. We compare: EBA, EBA_{plain}, AD and TM-align. We included only the sequence pairs for which TM-align was able to generate an alignment. In the left plot: on the x-axis we show the product of the length of the two sequences, while on the y-axis the computation time. In the one on the right we show the same thing, but in logarithmic scale, in order to appreciate the difference with the AD. The computation time includes only the generation of the similarity matrix and the computation of the alignment; not the per-residue embedding generation.

Table S3. Average computation times for PISCES's pairs. We compare: EBA, EBA_{plain}, AD and TM-align, including only the sequence pairs for which TM-align was able to generate an alignment.

	EBA	EBA_{plain}	AD	TM-align
average time (s)	0.02	0.02	$7 \cdot 10^{-5}$	0.17

3. FLEXIBLE DOMAIN IDENTIFICATION

Compared to the TM score, the method under consideration offers several advantages. For example, the TM score metric relies on rigid superpositions, which can limit similarity detection due to structural flexibility. For instance, as illustrated in Figure S2, two structures that undergo a hinge movement may receive a low TM score, whereas the EBA method can still accurately identify the similarity between the corresponding sequences.

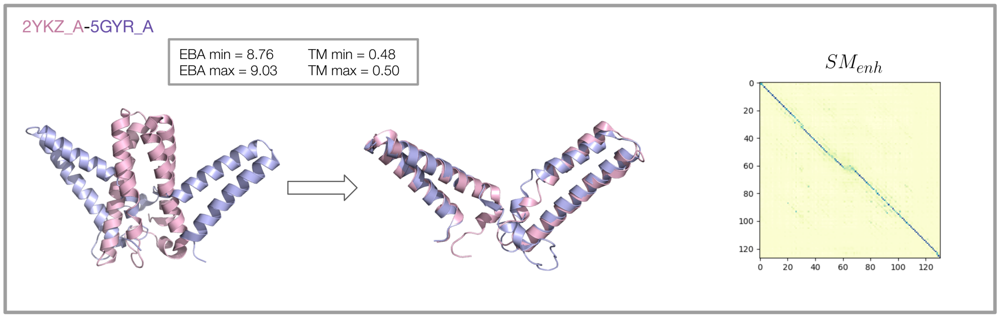

Fig. S2. Outlier of the EBA classification in the PISCES analysis. These proteins share the same domains but with a different relative orientation. Notably, the EBA method was able to capture the structural similarity between these proteins, whereas the TM score failed to do so.

4. PROTEIN LANGUAGE MODELS COMPARISON

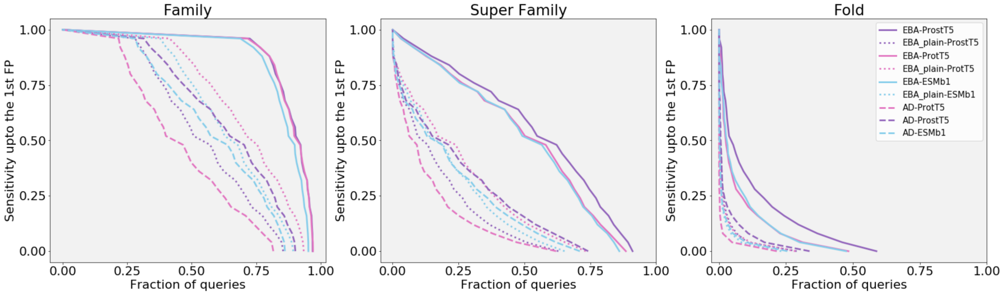

Fig. S3. Cumulative sensitivity distribution for the annotation transfer analysis on the SCOPe40 database for: family, super family and fold. The sensitivity is computed as the area under the ROC curve up to the first FP. With TPs being matches within the same group and FPs being matches between different folds. We report the performances of EBA, EBA$_{plain}$ and AD for the following protein language models: ProstT5 [3], ProtT5 [1], ESM-1b [9].

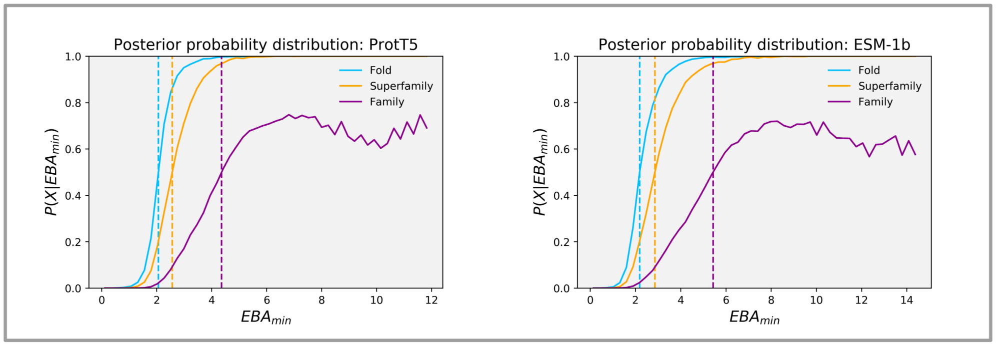

Fig. S4. Posterior probability of belonging to the same group in the transfer annotation analysis on the SCOPe40 dataset. The posterior probabilities are computed using EBA$_{min}$ with ProtT5 and ESM-1b as underling language models.

5. HOMSTRAD ALIGNMENT QUALITY

Table S4. Alignment quality sensitivity and precision for the HOMSTRAD [8] benchmark. With sensitivity being: TP residues in alignment/query length and precision being TP residues/alignment length. This values refer to the plot in Figure 3 panel B of the manuscript.

	Sequence-based			Structure-based				
	EBA	EBA_{plain}	MMseqs2	Foldseek	Foldseek-TM	TM-align	CLE-SW	DALI
Sensitivity	**0.859**	0.789	0.325	0.833	0.872	**0.877**	0.759	0.858
Precision	**0.861**	0.832	0.597	0.868	0.885	0.890	0.794	**0.913**

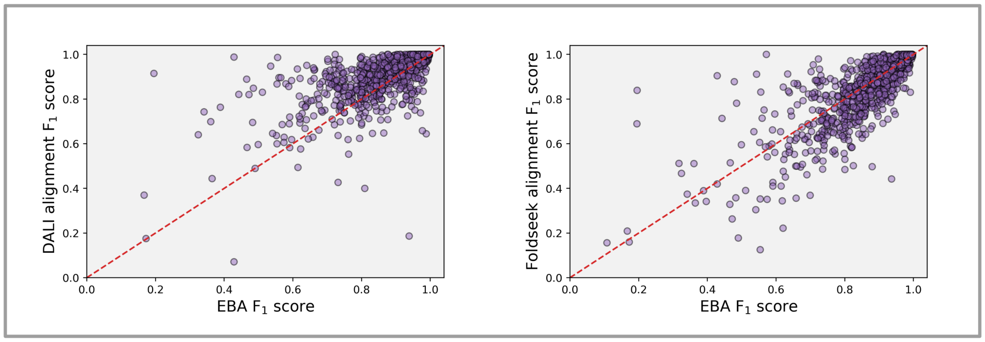

Fig. S5. Scatter plots for alignment quality comparison between EBA-ProstT5, DALI [4] and Foldseek [6]. For each element of the HOMSTRAD benchmark set we compare the F1 score generated with the different aligners. Notice that DALI failed in 46 of the alignments, we did not include those in the scatter plot.

6. SIGNAL ENHANCEMENT EXAMPLE

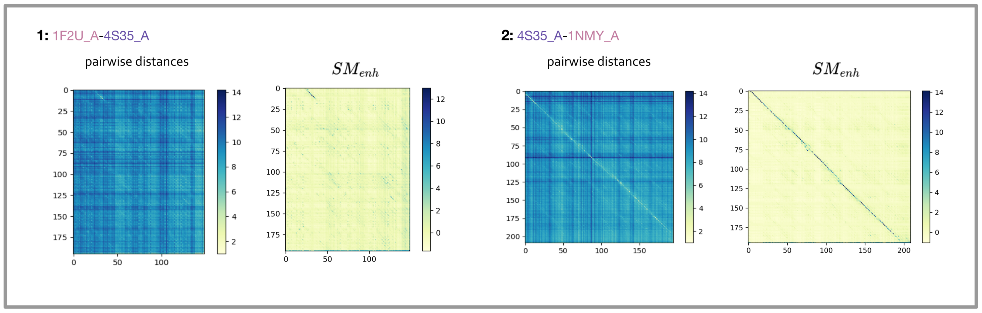

Fig. S6. Pairwise distance matrix and enhanced similarity matrix (SM_{enh}) of the two pairs of sequences shown in Figure 1 of the manuscript. This figure shows how the signal enhancement strengthens the signal for pair 2.

REFERENCES

1. Elnaggar, A., Heinzinger, M., Dallago, C., Rehawi, G., Wang, Y., Jones, L., Gibbs, T., Feher, T., Angerer, C., Steinegger, M., Bhowmik, D., Rost, B.: Prottrans: Toward understanding the language of life through self-supervised learning. IEEE Transactions on Pattern Analysis and Machine Intelligence **44**(10), 7112–7127 (2022). https://doi.org/10.1109/TPAMI.2021.3095381

2. Hamamsy, T., Morton, J.T., Berenberg, D., Carriero, N., Gligorijevic, V., Blackwell, R., Strauss, C.E.M., Leman, J.K., Cho, K., Bonneau, R.: Tm-vec: template modeling vectors for fast homology detection and alignment. bioRxiv (2022). https://doi.org/10.1101/2022.07.25.501437, https://www.biorxiv.org/content/early/2022/08/30/2022.07.25.501437

3. Heinzinger, M., Weissenow, K., Sanchez, J.G., Henkel, A., Steinegger, M., Rost, B.: Prostt5: Bilingual language model for protein sequence and structure. bioRxiv (2023). https://doi.org/10.1101/2023.07.23.550085, https://www.biorxiv.org/content/early/2023/07/25/2023.07.23.550085

4. Holm, L., Sander, C.: Protein structure comparison by alignment of distance matrices. Journal of Molecular Biology **233**(1), 123–138 (1993). https://doi.org/https://doi.org/10.1006/jmbi.1993.1489, https://www.sciencedirect.com/science/article/pii/S0022283683714890

5. Kaminski, K., Ludwiczak, J., Alva, V., Dunin-Horkawicz, S.: plm-blast – distant homology detection based on direct comparison of sequence representations from protein language models. bioRxiv (2022). https://doi.org/10.1101/2022.11.24.517862, https://www.biorxiv.org/content/early/2022/12/01/2022.11.24.517862

6. van Kempen, M., Kim, S.S., Tumescheit, C., Mirdita, M., Lee, J., Gilchrist, C.L., Söding, J., Steinegger, M.: Fast and accurate protein structure search with foldseek. Nature Biotechnology (2023). https://doi.org/https://doi.org/10.1038/s41587-023-01773-0

7. Madeira, F., Pearce, M., Tivey, A.R.N., Basutkar, P., Lee, J., Edbali, O., Madhusoodanan, N., Kolesnikov, A., Lopez, R.: Search and sequence analysis tools services from EMBL-EBI in 2022. Nucleic Acids Research **50**(W1), W276–W279 (04 2022). https://doi.org/10.1093/nar/gkac240, https://doi.org/10.1093/nar/gkac240

8. Mizuguchi, K., Deane, C.M., Blundell, T.L., Overington, J.P.: Homstrad: A database of protein structure alignments for homologous families. Protein Science **7**(11), 2469–2471 (1998). https://doi.org/https://doi.org/10.1002/pro.5560071126, https://onlinelibrary.wiley.com/doi/abs/10.1002/pro.5560071126

9. Rives, A., Meier, J., Sercu, T., Goyal, S., Lin, Z., Liu, J., Guo, D., Ott, M., Zitnick, C.L., Ma, J., Fergus, R.: Biological structure and function emerge from scaling unsupervised learning to 250 million protein sequences. Proceedings of the National Academy of Sciences **118**(15), e2016239118 (2021). https://doi.org/10.1073/pnas.2016239118, https://www.pnas.org/doi/abs/10.1073/pnas.2016239118

10. Steinegger, M., Meier, M., Mirdita, M., Vöhringer, H., Haunsberger, S.J., Söding, J.: Hh-suite3 for fast remote homology detection and deep protein annotation. BMC bioinformatics **20**(1), 1–15 (2019)
